# International Society of Ultrasound in Obstetrics and Gynecology (ISUOG) - the propagation of knowledge in ultrasound for the improvement of OB/GYN care worldwide: experience of basic ultrasound training in Oman

**DOI:** 10.1186/s12909-019-1866-6

**Published:** 2019-11-21

**Authors:** Nikolaos Vrachnis, Aris T. Papageorghiou, Caterina M. Bilardo, Alfred Abuhamad, Ann Tabor, Titia E. Cohen-Overbeek, Eleni Xilakis, Flora Mates, Sarah P. Johnson, Jon Hyett

**Affiliations:** 1 National and Kapodistrian University of Athens, Health Sciences School, Medical School, 124B Vasilissis Sophias Ave, 115 26 Athens, Greece; 2 St George’s NHS Foundation Trust Teaching Hospitals, St George’s Medical School, University of London, London, UK; 3 Department of Obstetrics and Prenatal Diagnosis Amsterdam University Medical Centres, Amsterdam and University Medical Centre Groningen, University van Groningen, Groningen, The Netherlands; 4 Department of Obstetrics and Gynecology, Eastern Virginia Medical School, Norfolk, VA USA; 5 Obstetrics, Copenhagen University Hospital, Rigshospitalet, Copenhagen, Denmark; 6 Department of Obstetrics & Gynaecology, Division of Obstetrics and Prenatal Medicine, Erasmus MC, University Medical Center Rotterdam, Rotterdam, The Netherlands; 7 ISUOG, London, UK; 8 RPA Women and Babies, Royal Prince Alfred Hospital, Camperdown, NSW Australia

**Keywords:** ISUOG, Ultrasound, Obstetrics and gynecology, Medical education

## Abstract

**Background:**

The aim of this study is to evaluate effectiveness of a new ISUOG (International Society of Ultrasound in Obstetrics and Gynecology) Outreach Teaching and Training Program delivered in Muscat, Oman.

**Methods:**

Quantitative assessments to evaluate knowledge and practical skills were administered before and after an ultrasound course for sonologists attending the ISUOG Outreach Course, which took place in November, 2017, in Oman. Trainees were selected from each region of the country following a national vetting process conducted by the Oman Ministry of Health. Twenty-eight of the participants were included in the analysis. Pre- and post-training practical and theoretical scores were evaluated and compared.

**Results:**

Participants achieved statistically significant improvements, on average by 47% (*p* < 0.001), in both theoretical knowledge and practical skills. Specifically, the mean score in the theoretical knowledge test significantly increased from 55.6% (± 14.0%) to 81.6% (± 8.2%), while in the practical test, the mean score increased from 44.6% (± 19.5%) to 65.7% (± 23.0%) (*p* < 0.001). Performance was improved post-course among 27/28 participants (96.4%) in the theoretical test (range: 14 to 200%) and among 24/28 (85.7%) trainees in the practical skills test (range: 5 to 217%).

**Conclusion:**

Application of the ISUOG Basic Training Curriculum and Outreach Teaching and Training Course improved the theoretical knowledge and practical skills of local health personnel. Long-term re-evaluation is, however, considered imperative to ascertain and ensure knowledge retention.

## Background

Ultrasound relies heavily on the technical skills of providers and effective training is essential to ensure consistently high-quality care. However, there is still a lack of standardized training for ultrasound in obstetrics and gynecology [1]. Recent surveys of trainees across the USA and Europe report widespread variability in availability, quality, and methods of training and assessment, both within and across countries [2]. As ultrasound equipment has become more affordable, its use (particularly in low and middle income countries) has also increased. This has raised additional challenges around implementation and personnel capacity and the demand for education and training is greater than ever [3].

The International Society of Ultrasound in Obstetrics and Gynecology (ISUOG) has been recognized, since its establishment in 1991, as a provider of high-quality medical education; it supports individuals and health services in obstetric and gynecological ultrasound. Among other initiatives, ISUOG is developing and providing systematic theoretical and practical training based on its basic training recommendations [4]. In particular, it is expanding educational activities globally through on-site, standardized, basic training courses. Since 2008, ISUOG has run nine such training courses and outreach programs in Haiti, Somaliland, Sudan, Oman, Ghana, Mongolia, Myanmar, Papua New Guinea, and Lebanon, with the aim of providing on-site education to local health personnel [5]. These training programs are contributing to ISUOG’s long-term vision that every woman in the world has access to ultrasound, that every scan provider is competent and that the diagnosis of obstetric and gynecologic conditions is effective so that women’s health outcomes improve [6, 7]. ISUOG is, moreover, carrying out research to validate its educational activities and demonstrate the effectiveness of the range of training programs offered [8]. ISUOG’s curriculum development and courses around the world are now sufficiently well established to allow scientific validation of these educational initiatives and determine their effectiveness. The aim of the study was to evaluate the learning outcomes of ISUOG’s Outreach Course.

## Methods

The study took place during an outreach training program for obstetric and gynecologic ultrasound in Muscat, Oman, from 5th to 9th November, 2017. The Omani government had previously selected 30 trainees to become trainers in their own regions and approved the study. We collected information and assessed learning competency, short-term retention, and adaptation to change of practice. Quantitative assessment to evaluate obstetric knowledge and practical skills was administered to sonologists before and after the training program. Twenty-eight of the trainees attended the course and were included in the analysis. All participants provided written informed consent and were assigned identification numbers to ensure confidentiality.

We administered multiple choice questions, created for this purpose by senior clinicians for ISUOG, to assess the theoretical background of participants. In order to evaluate the efficacy of the course, the performance of the 28 trainees, who answered 19 theoretical questions to determine their original level of knowledge, was analyzed. The content of these questions involved basic theoretical knowledge in obstetrical ultrasound pertinent to fetal anatomy, biometry, and pathology, as well as the techniques for performing fetal ultrasound. These 19 questions represented important learning points from all lectures delivered during the 5-day course. After the completion of the theoretical training, physicians were required to answer a double number of questions (40 multiple choice questions, including the 19 questions that were administered to them at the outset of the training program). These extra questions were from the same lectures as the pre-course questions and explored further the theoretical knowledge acquired through the course.

The practical skills component of the program was similarly evaluated using pre-and post-training sets, in accordance with current consensus criteria on how to evaluate ultrasound competence [9]. Assessment of practical skills was performed in a clinical setting with patients. At the beginning of the practical training session, each trainee was asked to display their current level of ultrasound skills by demonstrating how they would capture and evaluate fetal images and biometric measurements as well as transvaginal gynecological scan images. Trainees were asked to obtain the following images: head and abdominal circumference, femur length, placental localization including distance of placenta from internal cervical os, four-chamber fetal heart view and longitudinal section of uterus, and ovarian morphology in the obstetrical and the transvaginal ultrasound. Each training day comprised a 4-h theoretical session made up of lectures, which was followed by an equal time practical training in patients, during which participants were required to practice, among other skills, sliding, rotation, dipping, and angling transducer motions, as well as basic machine “knobology”. The training course involved a series of hands-on sessions relevant to the three trimesters of pregnancy and included the assessment of fetal lie and presentation, viability, number of fetuses, placenta location, amniotic fluid volume, and fetal biometry. The trainees practiced on volunteer women who provided consent. Each trainee was asked to produce a second copy of the images on the last day of practical training; one copy was kept with the patient notes and the other was used for practical assessment of the trainees by the trainer.

Pre- and post-training practical scores were evaluated according to the Objective Structured Assessment of Ultrasound Skills (OSAUS) on a scale of 1 to 5: this involves four elements concerning the images obtained, including applied knowledge of ultrasound equipment, image optimization, systematic examination, and interpretation of images (Additional file 1: Table S1 and Additional file 2: Table S2) [4].

The collected data were analyzed using MedCalc Statistical Software version 12.7.7 (2013 MedCalc Software bvba, Ostend, Belgium). Quantitative variables were expressed as mean ± standard deviation (SD). The paired t-test was applied to detect differences between pre- and post-course variables after normality verification using the Kolmogorov-Smirnov test. Qualitative variables were expressed as numbers and percentages, and analysis was performed with the chi-square or Fisher’s exact tests, as appropriate. *P* < 0.05 was considered statistically significant.

## Results

All trainees taking part in the Oman course were female, as male doctors rarely if ever work in this field in Oman [10], the Oman Medical Specialty Board having recorded no male residents graduating in this specialty between 2006 and 2015. Table 1 depicts the background characteristics of the trainees. The average age was 38 years, with a range of 31–47 years. Nine trainees (30%) had been practicing ultrasound for between 5 and 10 years before the course, 11% for 3–5 years, and 18% for 0–3 years. The remaining trainees did not specify. On average, the trainees completed 38 obstetric scans per week (numbers ranging from 1 to 120). Very few trainees had performed gynecological ultrasound scans on a regular basis, if at all. Approximately half of the trainees who responded to the survey had received their ultrasound certificate before the course and five had formal training in the form of the official National Curriculum in Fetal Ultrasound. A small number of trainees reported having practiced ultrasound for over 5 years despite no formal training and no supervised clinical practice; others had no formal training, though some supervision.

**Table 1:** Background characteristics of trainees

Trainee age (range)	38 (31–47)
Profession	
Specialist in fetal medicine	4
Obstetrician gynecologist	3
Trainee in obstetrics and gynecology	1
Fetal medicine trainee	3
General practicioner	8
Practice years	
0–3 years	5
3–5 years	5
5–10 years	9
Scans per week (< 20)	8/19
Formal training	9/19
Logbook available	3/19

**Fig. 1:**
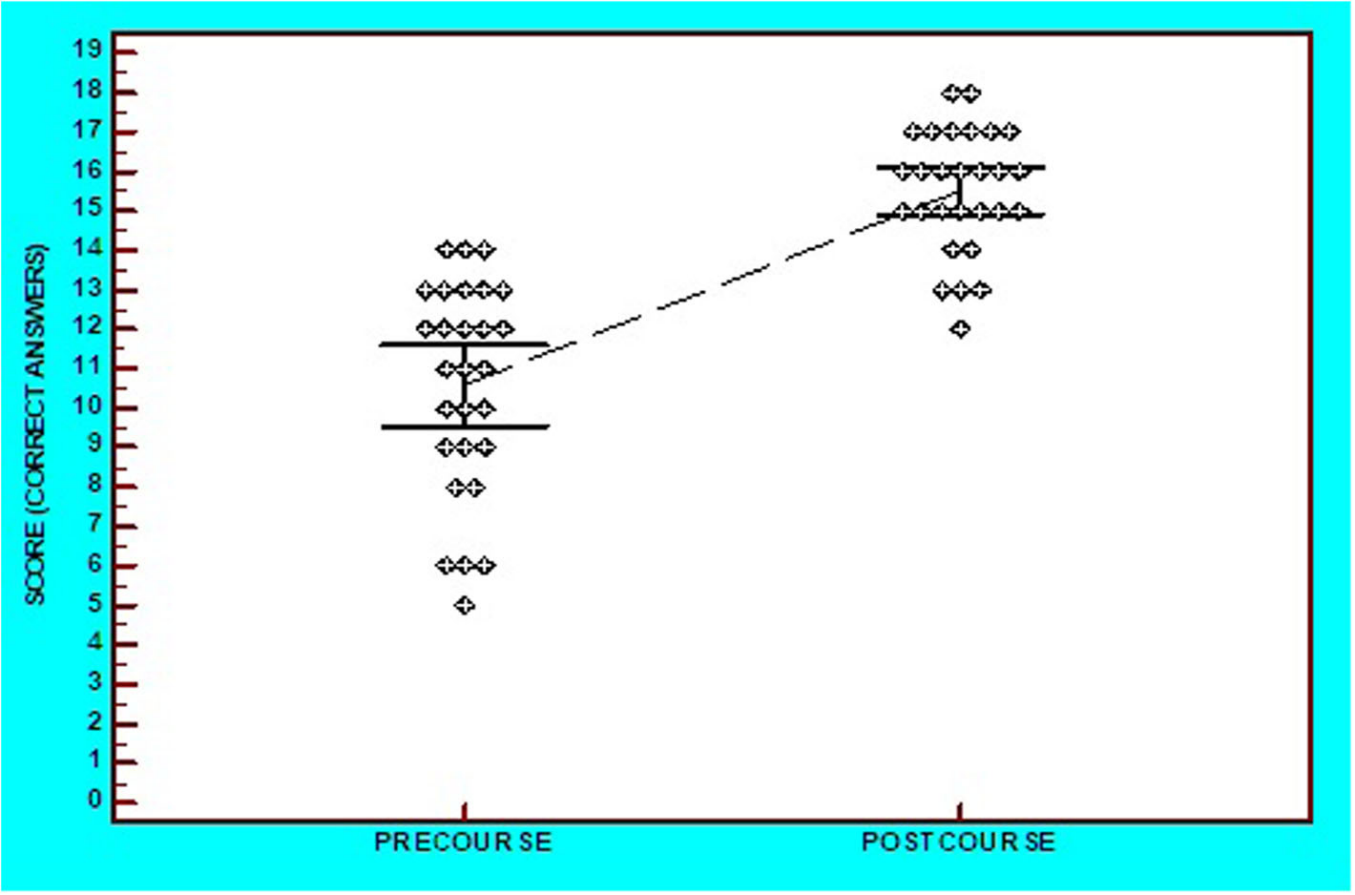
Dots-plot diagram with mean and 95%CI of pre- and post-theoretical course score

**Fig. 2:**
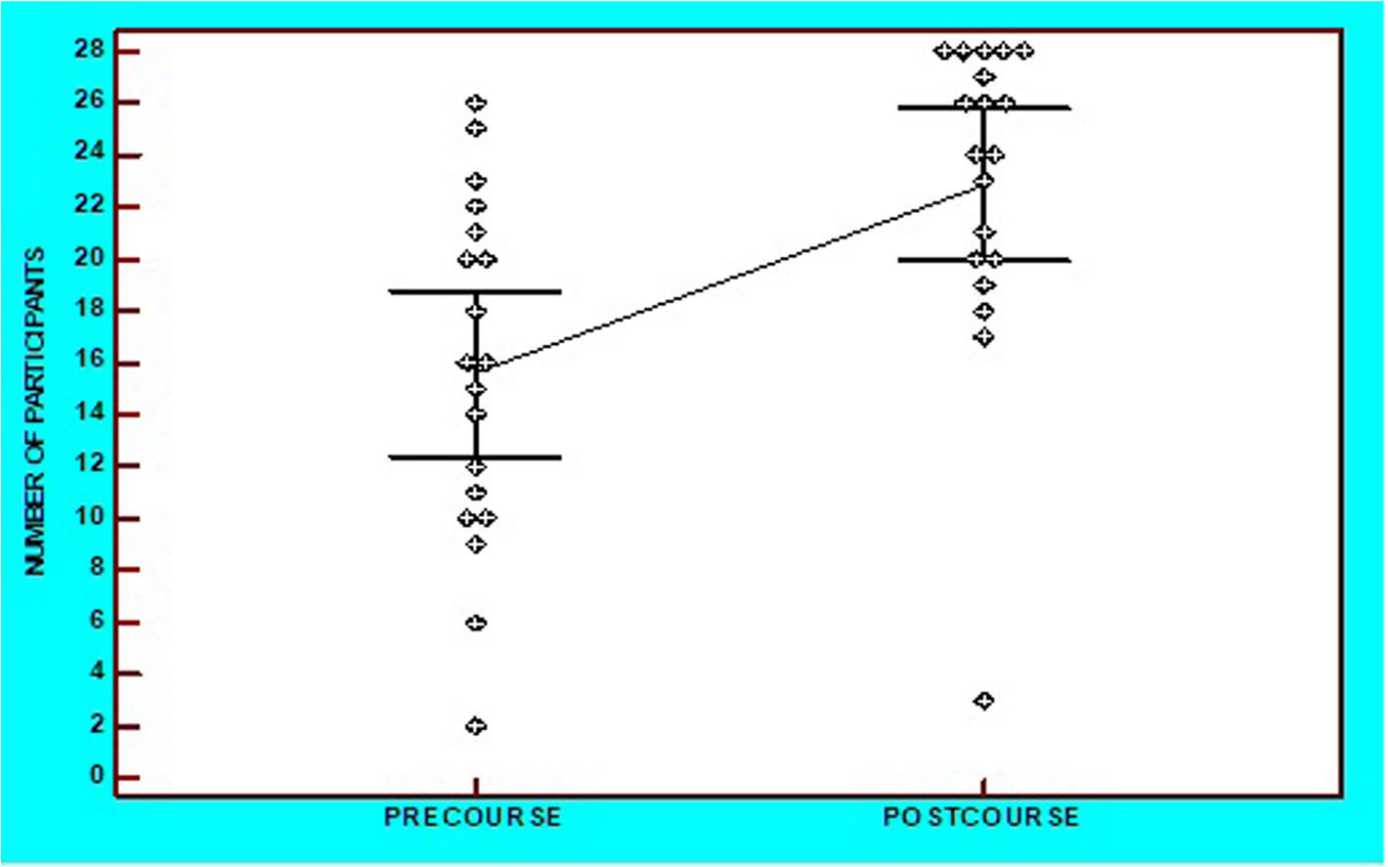
Dots-plot diagram with mean and 95%CI of the pre- and post-theoretical course comparison of questions by the number of trainees who answered correctly

From this study population of 30 trainees, 28 trainees were included in the analysis as two trainees did not attend the course. This analysis was composed of theoretical knowledge and practical skills assessment. Concerning the theoretical part, 19 questions were answered pre- and post-course. The score (number of correct answers per trainee) ranged from 26 to 74% in the pre-course test and from 63 to 95% in the post-course evaluation. There was a significant increase in the mean score, which increased from 56% (± 14%) to 82% (± 8%) (an increase of 47% (95%CI: 38 to 56%), *p* < 0.001). Twenty-seven out of the 28 trainees (96%) showed improvement, while in one case the performance worsened (Figs. 1 and 2).

In the pre-course test, the majority of trainees (54%) had a < 60% score, whereas all trainees (100%) scored > 60% post-course (OR = 65.4, 95%CI: 3.6 to 1177.2, *p* = 0.005). Furthermore, in the pre-course test, none (0%) of the trainees scored > 75%, whereas this figure was 79% post-course (OR = 197.3, 95%CI: 10.5 to 3691.9, *p* < 0.001). Finally, the post-course score distribution showed a notably smaller dispersion than the pre-course score (coefficient of variation: 25% vs 10%, respectively) (Fig. 1).

Pre-course, each question was answered correctly at an average rate of 56% ± 23%. This rose significantly in post-course scores by 82% ± 22%, a mean increase of 47% (95%CI: 33 to 60%, *p* < 0.001). The increase in the number of trainees answering correctly ranged from 6 to 217%. Pre-course, 36.8% of questions were answered correctly by trainees, while this proportion increased to 78.9% post-course (OR = 6.4, 95%CI: 1.5 to 27.2, *p* = 0.012). Only one question (5%) was answered correctly by ≥26 obstetricians pre-course, this increasing to 47% post-course (OR = 16.2, 95%CI: 1.8–147.1, *p* = 0.013) (Fig. 2).

**Fig. 3:**
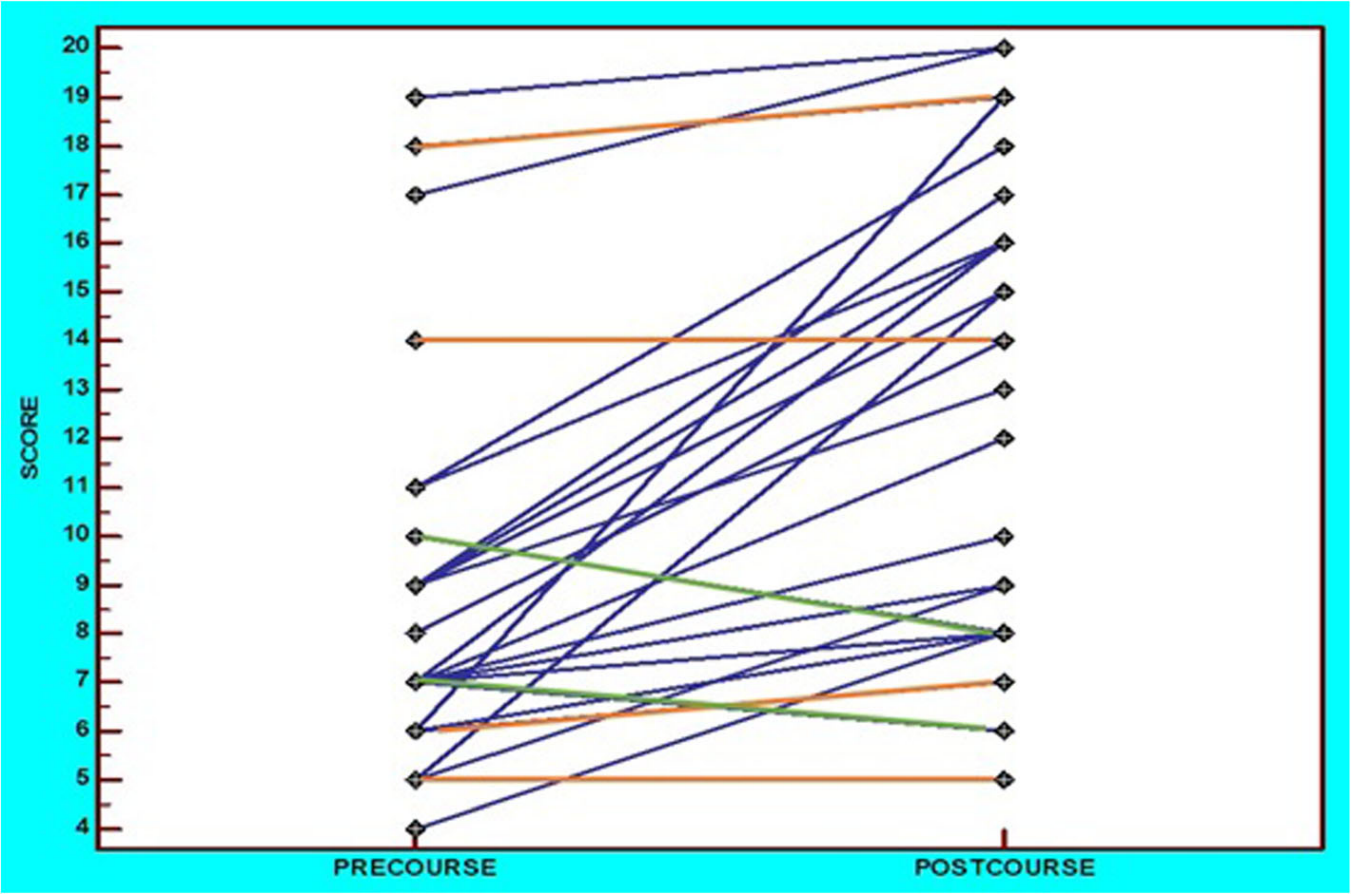
Dot and line diagram of the comparison of pre- and post-practical course scores. Scores that did not significantly differ between the pre- and post-practical courses are depicted in orange, while those that declined are presented in green

In the practical ultrasound skills test, scores ranged from 20 to 95% in the pre-course test and from 25 to 100% in the post-course evaluation. There was a significant increase in the score which increased from 45 to 66% (a mean increase of 47% *p* < 0.001). Eighty-six percent of the trainees improved their performance post-course by a range of 5–217%. Half of the trainees (14/28) improved by at least 50%, while four trainees improved by 100% or more. Only two trainees showed a worsening by 20 and 14%, while for two trainees, the score remained unchanged after the practical course. In the pre-course test, the majority of the trainees (71%) had a score of < 50%, whereas 69% of the trainees scored ≥50% post-course (OR = 5.3, 95%CI: 1.7 to 16.5, *p* = 0.004). Furthermore, in the post-course test, 64% of the trainees scored ≥60%, whereas this figure was 14% pre-course (OR = 10.8, 95%CI: 2.9 to 40.1, *p* < 0.001) (Fig. 3).

## Discussion

Several factors seem to create barriers to regular use of ultrasound in clinical practice. Among them, lack of appropriate training appears to be the most important issue. However, issues such as lack of equipment, equipment malfunction, and lack of maintenance of hardware seem to equally restrict dissemination of knowledge as well as retention of theoretical and practical skills that is required for optimal clinical practice [11]. In this study, we evaluated the performance of 28 trainee physicians in Oman with regard to the theoretical knowledge and practical skills acquired in obstetrical and gynecological ultrasound after a 5-day Outreach training course organized by ISUOG Outreach partners together with the Omani government; this was the first national ultrasound training conducted in this country. We showed that the course was associated with a remarkable improvement in post-course scores compared to pre-course, not only for theoretical but also practical components. This highlights the success of the course in fulfilling the training needs, as well as the goals of ISUOG.

Our study highlighted that there was significant heterogeneity in experience and inconsistency in prior training at baseline; this further validates our approach, as ISUOG’s Basic Training program ensures standardized training and ensures that all trainees have experience in both obstetric and gynecological ultrasound. As part of this, ISUOG also initiated the use of log books among trainees in order for each trainee to better record her experience obtained and practice performed throughout the year.

Before the course, the participants gave the appearance of forming a rather disparate group, showing a significant dispersion in the level of their theoretical knowledge. By the end of the training process, however, the participants, overall, gave evidence of having considerably improved their level of knowledge, while their differences in knowledge were remarkably decreased with respect to the theoretical subjects they had been taught. Identification of these differences leads to the conclusion that the interobserver discrepancy noted pre- and post-course could also be partly attributed to provider skills. These differences have already been described in the setting of advanced gynecological ultrasound [12]; however, it remains unclear whether the trainers’ ability to evaluate the skill of participants is enhanced following the completion of these courses.

The results of the practical course evaluation were equally impressive: before practical training, the trainees demonstrated their initial level of ultrasound skill by producing specific images in obstetric and gynecological ultrasound. After its completion, the trainees were asked to once again produce the specific images, and their pre- and post-course performance was evaluated based on the OSAUS scoring system (Additional file 2: Table S2). The average improvement of practical skills (47% (range 45–66%)) is also very encouraging and underlines the importance of this Outreach Program.

Despite the ubiquitous success of the project, two participants had lower scores following the completion of the practical component of the course. Obviously, the acquisition of practical skills may be slower in some trainees, but the aforementioned minimal decline that was shown in practical skills may be due to two incidental factors: the position of the baby during the final practical exam, which made it more technically demanding for the specific trainee, and the maternal habitus, since the exam took place in real pregnant women. These occurrences happen unexpectedly as the baby moves or stays still for prolonged periods and in unfavorable positions.

One limitation of our study is that we only assessed short-term retention of information. Data concerning trainees’ retention of the theoretical background are scarce in the literature and seem to be conflicting [13, 14]. While several methods have been suggested, including live lectures, podcasts, and e-learning activities, none of them has proved to be superior to the old teaching methods [14, 15]. In 2016, Hempel et al. demonstrated that the incorporation of an e-learning activity following the completion of a hands-on training session significantly increases knowledge retention and trainees’ satisfaction [16]. To this end, WhatsApp Messenger groups (https://en.wikipedia.org/wiki/WhatsApp) were established after the conclusion of this week of training between the designated trainer and the Omani trainees to ensure continuous communication for the next semester, which will enable ultrasound image and text exchange so that trainees’ questions can be answered; further work to assess these longer-term effects on retention of knowledge and skills is underway.

## Conclusion

The findings of our study show that the conduct of basic ultrasound training programs significantly improves both the theoretical and practical skills of trainees. The results of this analysis demonstrate that the basic training course in Oman, like many others previously organized in numerous other countries by ISUOG, successfully achieved the ISUOG goals and gives every appearance of serving its purpose for the propagation of physicians’ and midwives’ knowledge in ultrasound, with the ultimate goal of improving healthcare in the field of obstetrics and gynecology worldwide. Future research activities should evaluate the retention of skills over time and the successful implementation of ultrasound in prenatal care in order to ensure long-term sustainability of providing quality basic ultrasound examinations to women in low-resource settings.

## Supplementary information

**Additional file 1: Table S1.** Objective Structured Assessment of Ultrasound Skills (OSAUS.

**Additional file 2: Table S2.** OSAUS questionnaire for each investigated parameter.

## Data Availability

The data that support the findings of this study are available from ISUOG, but restrictions apply to the availability of these data, which were used under license for the current study and so are not publicly available. Data are however available from the authors upon reasonable request and with permission of ISUOG.

## Abbreviations

ISUOG: International Society of Ultrasound in Obstetrics and Gynecology
OSAUS: Objective Structured Assessment of Ultrasound Skills
SD: standard deviation

## References

[1] Leonardi M, Murji A, D’Souza R (2018). Ultrasound curricula in obstetrics and gynecology training programs. Ultrasound Obstet Gynecol.

[2] Alrahmani L, Codsi E, Borowski KS (2018). The current state of ultrasound training in obstetrics and gynecology residency programs. J Ultrasound Med.

[3] Kim ET, Singh K, Moran A, Armbruster D, Kozuki N (2018). Obstetric ultrasound use in low and middle income countries: a narrative review. Reprod Health.

[4] ISUOG (2014). Education committee recommendations for basic training in obstetric and gynecological ultrasound. Ultrasound Obstet Gynecol.

[5] Creatsas G, Vrachnis N (2006). Hospital visiting in obstetrics and gynecology: a tool for the advancement of training. Int J Gynaecol Obstet.

[6] Au K, Lam D, Garg N, Chau A, Dzwonek A, Walker B, Tremblay L, Boet S, Bould MD (2019). Improving skills retention after advanced structured resuscitation training: a systematic review of randomized controlled trials. Resuscitation.

[7] Abuhamad A, Minton KK, Benson CB, Chudleigh T, Crites L, Doubilet PM, Driggers R, Lee W, Mann KV, Perez JJ (2018). Obstetric and gynecologic ultrasound curriculum and competency assessment in residency training programs: consensus report. Ultrasound Obstet Gynecol.

[8] Tolsgaard MG, Chalouhi GE (2018). Use of simulators for the assessment of trainees’ competence: trendy toys or valuable instruments?. Ultrasound Obstet Gynecol.

[9] Tolsgaard MG, Todsen T, Sorensen JL, Ringsted C, Lorentzen T, Ottesen B, Tabor A (2013). International multispecialty consensus on how to evaluate ultrasound competence: a Delphi consensus survey. PLoS One.

[10] Mohamed NA, Abdulhadi NN, Al-Maniri AA, Al-Lawati NR, Al-Qasmi AM (2018). The trend of feminization of doctors’ workforce in Oman: is it a phenomenon that could rouse the health system?. Hum Resour Health.

[11] Shah S, Bellows BA, Adedipe AA, Totten JE, Backlund BH, Sajed D (2015). Perceived barriers in the use of ultrasound in developing countries. Crit Ultrasound J.

[12] Sladkevicius P, Valentin L (2015). Interobserver agreement in describing the ultrasound appearance of adnexal masses and in calculating the risk of malignancy using logistic regression models. Clin Cancer Res.

[13] Hempel D, Stenger T, Campo Dell” Orto M, Stenger D, Seibel A, Rohrig S, Heringer F, Walcher F, Breitkreutz R (2014). Analysis of trainees’ memory after classroom presentations of didactical ultrasound courses. Crit Ultrasound J.

[14] Hempel D, Sinnathurai S, Haunhorst S, Seibel A, Michels G, Heringer F, Recker F, Breitkreutz R (2016). Influence of case-based e-learning on students’ performance in point-of-care ultrasound courses: a randomized trial. Eur J Emerg Med.

[15] Florescu CC, Mullen JA, Nguyen VM, Sanders BE, Vu PQ (2015). Evaluating didactic methods for training medical students in the use of bedside ultrasound for clinical practice at a Faculty of Medicine in Romania. J Ultrasound Med.

[16] Hempel D, Haunhorst S, Sinnathurai S, Seibel A, Recker F, Heringer F, Michels G, Breitkreutz R (2016). Social media to supplement point-of-care ultrasound courses: the “sandwich e-learning” approach. A randomized trial. Crit Ultrasound J.

