# Supplementary material for: International Society of Ultrasound in Obstetrics and Gynecology (ISUOG) - the propagation of knowledge in ultrasound for the improvement of OB/GYN care worldwide: experience of basic ultrasound training in Oman
Source: BMC Med Educ. 2019 Nov 21;19:434. doi: 10.1186/s12909-019-1866-6 (PMC6873715; doi:10.1186/s12909-019-1866-6)
Supplement: Supplementary file 1 — Additional file 1: Table S1. Objective Structured Assessment of Ultrasound Skills (OSAUS. [file 12909_2019_1866_MOESM1_ESM.docx]

| **Additional file 1: Table S1. Objective Structured Assessment of Ultrasound Skills (OSAUS)**  Each trainee is rated from 1-5 in all of the elements listed below when obtaining the images.   \| **1. Applied knowledge of ultrasound equipment**  Familiarity with the equipment and its functions, i.e. selecting probe, using buttons, and application of gel \| 1  Unable to operate equipment \| 2 \| 3  Operates the equipment with some experience \| 4 \| 5  Obviously familiar with operating the equipment and all of its functions \| \| --- \| --- \| --- \| --- \| --- \| --- \| \| **2. Image optimization**  Consistently ensuring optimal image quality by adjusting gain, depth, focus, frequency, etc. \| 1  Fails to optimize images \| 2 \| 3  Competent image optimization but not done consistently \| 4 \| 5  Consistent and excellent optimization of images \| \| **3. Systematic examination**  Consistently displaying systematic approach to the examination and presentation of relevant structures \| 1  Unsystematic approach. Unable to present most relevant structures. \| 2 \| 3  Displays some systematic approach and localizes most relevant structures \| 4 \| 5  Consistently displays systematic approach and presents all relevant structures \| \| **4. Interpretation of images**  Recognition of image pattern and interpretation of findings \| 1  Unable to interpret any findings \| 2 \| 3  Does not consistently interpret findings correctly \| 4 \| 5  Recognizes image patterns quickly and interprets findings correctly \|   **Total score:**__________________________  Evaluator:__________________________________________  Trainee:__________________________________________________  Images evaluated:_________________________________________________ |
| --- | --- | --- | --- | --- | --- | --- | --- | --- | --- | --- | --- | --- | --- | --- | --- | --- | --- | --- | --- | --- | --- | --- | --- | --- |
