# Supplementary material for: International Society of Ultrasound in Obstetrics and Gynecology (ISUOG) - the propagation of knowledge in ultrasound for the improvement of OB/GYN care worldwide: experience of basic ultrasound training in Oman
Source: BMC Med Educ. 2019 Nov 21;19:434. doi: 10.1186/s12909-019-1866-6 (PMC6873715; doi:10.1186/s12909-019-1866-6)
Supplement: Supplementary file 2 — Additional file 2: Table S2. OSAUS questionnaire for each investigated parameter. [file 12909_2019_1866_MOESM2_ESM.docx]

| **Additional file 2: Table S2. OSAUS practical assessment questionnaire** | | | |
| --- | --- | --- | --- |
| **Pre-course** | | **Post-course** | |
| Name: |  | Name: |  |
| Trainee number: |  | Trainee number: |  |
| Supervisor: |  | Supervisor: |  |
| **Image #1** | **Taken by trainee (Yes/No)** | **Image #1** | **Taken by trainee (Yes/No)** |
| **Image & Features** |  | **Image & Features** |  |
| **Head circumference / BPD** |  | **Head circumference / BPD** |  |
| Appropriate symmetrical axial section |  | Appropriate symmetrical axial section |  |
| Demonstration of the cavum septum pellucidum, thalamus, and choroid plexi |  | Demonstration of the cavum septum pellucidum, thalamus, and choroid plexi |  |
| Good image size (head = >75% of screen) |  | Good image size (head = >75% of screen) |  |
| Accurate calliper placement for BPD (outer-inner) |  | Accurate calliper placement for BPD (outer-inner) |  |
| Accurate calliper placement HC (outer border of cranium) |  | Accurate calliper placement HC (outer border of cranium) |  |
| Trainer’s note: |  | Trainer’s note: |  |
| **Image #2** | **Taken by trainee (Yes/No)** | **Image #2** | **Taken by trainee (Yes/No)** |
| **Image & Features** |  | **Image & Features** |  |
| **Abdominal circumference** |  | **Abdominal circumference** |  |
| Appropriate axial section (not oblique) |  | Appropriate axial section (not oblique) |  |
| Good image size |  | Good image size |  |
| Demonstration of umbilical vein and stomach |  | Demonstration of umbilical vein and stomach |  |
| Demonstration of transverse section of spine and single ribs |  | Demonstration of transverse section of spine and single ribs |  |
| Accurate calliper placement / measurement with ellipse tool |  | Accurate calliper placement / measurement with ellipse tool |  |
| Trainer’s note: |  | Trainer’s note: |  |
| **Image #3** | **Taken by trainee (Yes/No)** | **Image #3** | **Taken by trainee (Yes/No)** |
| **Image & Features** |  | **Image & Features** |  |
| **Femur Length** |  | **Femur Length** |  |
| Good image size |  | Good image size |  |
| Femur shown horizontally |  | Femur shown horizontally |  |
| Both end points clearly imaged |  | Both end points clearly imaged |  |
| Completely encircled by muscular / subcuticular tissue of the thigh |  | Completely encircled by muscular / subcuticular tissue of the thigh |  |
| Accurate calliper placement on end points of long bone |  | Accurate calliper placement on end points of long bone |  |
| Trainer’s note: |  | Trainer’s note: |  |
| **Image #4** | **Taken by trainee (Yes/No)** | **Image #4** | **Taken by trainee (Yes/No)** |
| **Image & Features** |  | **Image & Features** |  |
| **Cervix / placental localization** |  | **Cervix / placental localization** |  |
| A longitudinal image of the lower segment of the uterus |  | A longitudinal image of the lower segment of the uterus |  |
| Panoramic view |  | Panoramic view |  |
| Demonstrating the internal cervical os |  | Demonstrating the internal cervical os |  |
| Demonstrating the position of the placenta |  | Demonstrating the position of the placenta |  |
| Measurement from leading edge of placenta to internal cervical os |  | Measurement from leading edge of placenta to internal cervical os |  |
| Trainer’s note: |  | Trainer’s note: |  |
